# The Space From Heart Disease Intervention for People With Cardiovascular Disease and Distress: A Mixed-Methods Study

**DOI:** 10.2196/resprot.4280

**Published:** 2015-07-01

**Authors:** Elizabeth Alexandra Barley, Abigail Clifton, Geraldine Lee, Ian J Norman, David O'Callaghan, Karen Tierney, Derek Richards

**Affiliations:** ^1^ Post Graduate Research Department Florence Nightingale Faculty of Nursing and Midwifery King's College London London United Kingdom; ^2^ SilverCloud Health The Priory John Street West Dublin 8 Ireland; ^3^ School of Psychology Trinity College Dublin Ireland

**Keywords:** Internet, depression, anxiety, well-being, cognitive behavioral therapy, behavioral therapy, self-management, cardiovascular disease, online interventions

## Abstract

**Background:**

Poor self-management of symptoms and psychological distress leads to worse outcomes and excess health service use in cardiovascular disease (CVD). Online-delivered therapy is effective, but generic interventions lack relevance for people with specific long-term conditions, such as cardiovascular disease.

**Objective:**

To develop a comprehensive online CVD-specific intervention to improve both self-management and well-being, and to test acceptability and feasibility.

**Methods:**

Informed by the Medical Research Council (MRC) guidance for the development of complex interventions, we adapted an existing evidence-based generic intervention for depression and anxiety for people with CVD. Content was informed by a literature review of existing resources and trial evidence, and the findings of a focus group study. Think-aloud usability testing was conducted to identify improvements to design and content. Acceptability and feasibility were tested in a cross-sectional study.

**Results:**

Focus group participants (n=10) agreed that no existing resource met all their needs. Improvements such as "collapse and expand" features were added based on findings that participants’ information needs varied, and specific information, such as detecting heart attacks and when to seek help, was added. Think-aloud testing (n=2) led to changes in font size and design changes around navigation. All participants of the cross-sectional study (10/10, 100%) were able to access and use the intervention. Reported satisfaction was good, although the intervention was perceived to lack relevance for people without comorbid psychological distress.

**Conclusions:**

We have developed an evidence-based, theory-informed, user-led online intervention for improving self-management and well-being in CVD. The use of multiple evaluation tests informed improvements to content and usability. Preliminary acceptability and feasibility has been demonstrated. The Space from Heart Disease intervention is now ready to be tested for effectiveness. This work has also identified that people with CVD symptoms and comorbid distress would be the most appropriate sample for a future randomized controlled trial to evaluate its effectiveness.

## Introduction

Cardiovascular diseases (CVDs) are the leading cause of disability and mortality worldwide [1]. Atherosclerosis is responsible for a large proportion of CVD, including coronary heart disease (CHD). In 2010, heart attacks were responsible for 80,000 deaths [2]. CHD is a chronic condition that affects approximately 3.5% of the UK population [3]. Long-term conditions (LTCs), such as CHD, can be managed but not cured. Making healthy lifestyle choices, such as stopping smoking, eating healthily, drinking alcohol only in moderation, and being active, are important for physical health outcomes as well as for quality of life [4]. Self-management, whereby people take responsibility for their own health and well-being through staying fit and healthy, taking action to prevent illness and accidents, using medicines effectively, treating minor ailments appropriately, and seeking professional help when necessary, is key [5]. However, self-management can be compromised in the presence of comorbid depression and anxiety [6], which is more prevalent in CVD patients than in the general population [7,8].

Symptoms such as chest pain, palpitations, breathlessness, and fatigue are common in CHD and other CVD-related conditions. These symptoms may have a cardiac origin, but patients with CHD also experience symptoms when no cardiac cause can be found [9-12] and often no further physical treatment is available. In a recent cohort study (n=803) of primary care patients living with CHD, 44% reported current chest pain despite receiving treatment for their CHD [13]. Comorbid depression and anxiety can also exacerbate the perceived severity of physical symptoms such as chest pain, palpitations, breathlessness, and fatigue [6]; this often leads to increased primary care [14] and emergency department [15] attendance.

Access to effective psychological treatment for depression and anxiety for people with CVD is limited, such as cognitive behavioral therapy (CBT) delivered by the UK government’s Improving Access to Psychological Therapy (IAPT) services, waiting lists can be long, and patients with physical health problems may be unwilling or unable to attend psychological therapy [6,16]. There is evidence that CBT is effective in the management of psychological symptoms of CVD [17], but it is not widely available. In the United Kingdom, the government’s IAPT long-term conditions/medically unexplained symptoms (LTC/MUS) Pathfinder Project was established in 2012 to improve access to psychological therapies for people with LTCs and medically unexplained symptoms (MUS). The management of LTC/MUS using behavioral and cognitive behavioral therapy is being tested in a small sample of IAPT services; a recent interim report suggests some clinical and cost benefits, although problems with data collection mean that findings have to be interpreted with caution [18].

Online-delivered interventions can be a low-cost and nonstigmatizing way of delivering therapy and self-management support, and they are easily accessed. Online-delivered therapy is effective for psychological distress [19], although low levels of engagement can lead to poor patient outcomes [20]. Furthermore, established generic computerized cognitive behavioral therapy packages, such MoodGym [21] and Beating the Blues [22], may not be acceptable for people with comorbid physical health problems [23]. A solution that includes disease-specific content is needed to maximize the health benefit.

Our searches of electronic reference databases suggests that interest in Internet-delivered interventions for patients with long-term conditions including CHD is increasing, though data on their effectiveness are scarce. For instance, the E-Rehabilitation intervention [24] includes information regarding cardiac rehabilitation, a discussion forum, and an activity calendar. In a randomized controlled trial (RCT), intervention group participants received tailored content based on models of health behavior through the website and mobile text messages. Short-term results in 69 patients indicated that at 1- and 3-months posttreatment there was a higher median level of physical activity in the active treatment group compared to the control group; this was statistically significant at 3 months. No significant statistical differences were found between the treatment and control groups on self-efficacy, social support, anxiety, or depression [25]. This intervention was not designed specifically to address comorbid psychological distress.

A number of protocols for trials of online CHD self-management support have been published. The InterHerz project from Switzerland [26] will provide an established intervention for depression treatment (Deprexis) to cardiac patients. However, this intervention is not CHD specific, so may lack relevance to this population. In the United Kingdom, a comparison of usual care—National Health Service (NHS)—with usual care plus access to the NHS Helpline service to reduce levels of cardiac risk factors [27] is planned. In Canada, a trial of e-counselling text messages for adherence to lifestyle change in people with hypertension [28] is underway. Neither intervention appears to offer tailored, comprehensive support for both CHD self-management and comorbid psychological distress.

Therefore, it appears that while some interventions are used to address the physical and lifestyle management of CHD and others address psychological distress in this population, there are no products that take a holistic approach to address both self-management and psychological distress in CHD. Informed by the Medical Research Council (MRC) guidance for the development of complex interventions [29], we have developed such a holistic online intervention: Space from Heart Disease. The development phase of the MRC guidelines includes "identifying the evidence base," "identifying or developing theory," and "modelling process and outcomes"; reporting of this phase is important as it informs later implementation and evaluation [29].

Space from Heart Disease uses an existing platform developed by SilverCloud Health which has been shown to be effective for delivering CBT for depression and anxiety [30-32] and which is currently used in 11 NHS trusts. The anxiety and depression platform has demonstrated a three-fold increase in user engagement and a three-fold decrease in user dropout rates compared to other online therapeutic products through the use of a trained supporter who can provide online, timely, personalized feedback [33,30]. This paper describes the development of CVD-specific content, informed by current evidence and by a focus group with CHD patients, and reports preliminary findings concerning the intervention’s acceptability and feasibility. Development of the supporter role will be the focus of future work.

## Methods

### Overview

**Figure 1:**
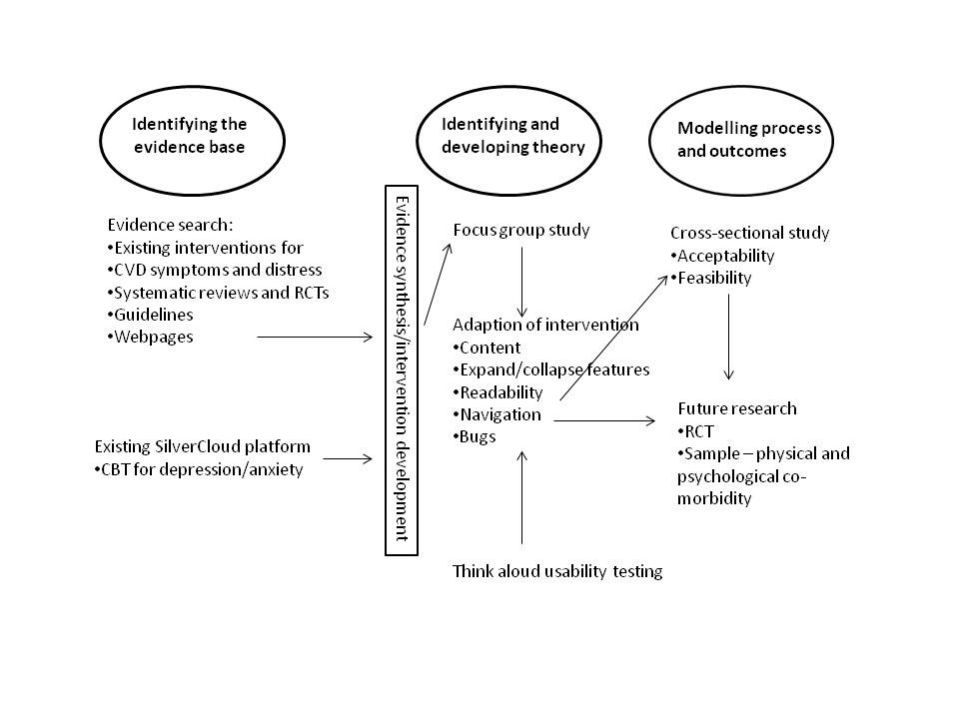
Medical Research Council development stage of Space from Heart Disease.

This work comprises four phases: development of the intervention and three experimental studies. The relation of this work to the MRC Framework for the Development of Complex Interventions [29] is depicted in Figure 1. Ethical approval for the work was provided by the Psychiatry, Nursing and Midwifery Research Ethics Subcommittee at King’s College London (KCL) (PNM/13/14-135). The work was funded by NHS England as part of their Small Business Research Initiative scheme.

### Phase 1: Development of the Intervention

We identified existing evidence-based interventions for common physical (ie, chest pain, palpitations, breathlessness, fatigue) and associated psychological (ie, depression, anxiety, stress, subclinical distress) symptoms of CVD.

We searched electronic databases—Cochrane Database of Systematic Reviews, Database of Abstracts of Reviews of Effects, Embase, Medline, and PsycINFO—for relevant systematic reviews and randomized controlled trials of interventions for CHD symptoms or distress. We also obtained relevant resources currently used in, or recommended for use by, Southwark IAPT service; this service is participating in the Pathfinder study [18]. National Institute for Health and Care Excellence (NICE) guidelines and high-quality websites, including NHS Choices [34] and the British Heart Foundation (BHF) [35], were reviewed.

This information was then used to adapt the existing SilverCloud intervention for psychological distress so that it was relevant to people with CVD and to develop new CVD-specific content (Figure 1). The intervention content was developed by the research team in conjunction with SilverCloud Health. The research team includes the following: a health psychologist, practitioner psychologist and nurse (EB), a cognitive behavior therapist and nurse (IN), a cardiac nurse (GL), and a research assistant who previously worked as an assistant psychologist within IAPT (AC). The SilverCloud team included people with expertise in developing and delivering online interventions (DR, KT, DOC).

### Phase 2: Focus Group Study

#### Participants

Participants were recruited via the British Heart Foundation. An email providing details of the project and the researcher’s contact details was sent by the BHF support group coordinator to the primary contact for support groups based in South East London. The primary contact for each group was asked to forward the email to their members. The email-recruitment-of-volunteers system at King’s College London was also used and a study recruitment poster was placed in a local supermarket.

Those interested in participating were screened to ensure they met the inclusion criteria, which were the following: aged 18 years or over with a self-reported diagnosis of CVD (ie, a diagnosis of one or more of coronary heart disease, atrial fibrillation, stroke, hypertension, diabetes, chronic kidney disease, and/or peripheral arterial disease), able to give informed consent, and able to read and understand English.

#### Data Collection

A focus group was conducted in the meeting room in the National Institute for Health Research (NIHR)/Wellcome Clinical Research Facility at King’s College Hospital. Participants were compensated with a payment of a £20 Love to Shop voucher and travel expenses were reimbursed. The group was facilitated by EB using a topic guide (see Multimedia Appendix 1) informed by literature review and expert opinion—study team and collaborators—to guide the discussion. Topics included current coping strategies (ie, their own or, if not personally relevant, any strategies they are aware of) for chest pain, fatigue, breathlessness, and distress; barriers and facilitators to healthy living (ie, diet, exercise, alcohol); and medication adherence. An example SilverCloud intervention (ie, the online intervention for depression currently being used in 11 NHS IAPT services) was demonstrated by a member of the SilverCloud team and participants were asked whether they thought a similar intervention would be useful to them and, if so, what adaptations would be needed. Example patient stories for use in the intervention were also discussed.

Participants were encouraged to give their opinion so that a wide range of views was understood; it was reiterated that a consensus was not being sought and that all opinions were important. The focus group was audiotaped with consent and transcribed verbatim. Anonymized field notes were made by AC and GL. At the beginning of the focus group session, participants completed a brief demographic questionnaire (ie, age, ethnicity, diagnoses, employment status and occupation, and highest academic achievement). They were also asked to provide their consent to be contacted to take part in the future development studies.

#### Data Analysis

The focus group transcript was read by two of the authors (EB and AC) to identify key themes. Active searching for disconfirming examples, for instance, statements where participants contradict or disagree with one another, was undertaken. The two researchers compared notes and reached consensus on the themes. Field notes were also considered. Themes were agreed upon by discussion within the multidisciplinary research team. Adaptations were made to the intervention informed by the focus group data (see Figure 1).

### Phase 3: Think-Aloud Usability Testing

#### Participants

The most vocal member of the above focus group was asked to return to test the intervention once changes had been made based on feedback. This person was selected as the participant most likely to be able to articulate their actions and choices during the think-aloud test. We also recruited the first female to respond to the invitation to participate via the BHF.

#### Data Collection

The intervention was made available on a computer in a soundproofed interview room in the NIHR/Wellcome Clinical Research Facility at King’s College Hospital. Written consent to videotape the test was obtained from the participants. The participants were asked to spend up to one hour each exploring the online intervention in any way they wanted. Whilst doing so, they were asked to describe aloud what they were thinking and doing. The study research assistant (AC) attended the session in order to answer any questions and to make notes; EB watched the test via closed-circuit television (CCTV) and also made field notes.

#### Data Analysis

Audio-visual recordings were observed by the study team in conjunction with the anonymized notes; problems with the intervention or difficulties using it were noted to inform changes to the intervention (see Figure 1).

### Phase 4: Cross-Sectional Study

### Participants

People with CVD were recruited from the pool of participants who participated in studies above and who had given consent to be contacted for future studies. In addition, snowballing (ie, recruitment of people with CVD known to the study team) was employed. An additional inclusion criterion was access to an Internet-connected computer. Travel expenses and a £20 voucher were provided to participants.

#### Data Collection

Participants were given access to the adapted intervention (ie, the version including modifications made in response to findings of the above studies) via a password, which allowed access from any computer for 2 weeks. Data concerning patterns of usage of, and satisfaction with, the intervention, as well as clinical data were collected electronically via the secure SilverCloud system. All data were anonymized. Usage data collected over 2 weeks were as follows: number of sessions per user, time spent per session, and total time spent using the intervention. A session was defined as a period of 5 minutes or more of a user being logged onto the system. Log-ins for less than 5 minutes were also recorded. Session time estimation will always be an imperfect calculation, as users may be interrupted or take breaks within a session, and may not formally log out of the system. All user activity within the system, such as reading a content page, saving a journal entry, or updating an activity, was logged with a time stamp. Starting with the log entry of the user logging on, the total time was calculated by adding up the time that elapsed between each subsequent log record (in the same manner as popular Web analytics software). On its own, this will yield a result vulnerable to overestimation of session time, so to avoid counting periods where the user is not actively engaged with the system, any inactivity (ie, lack of "clicks" within content) longer than 30 minutes was counted as 1 minute. Any period of inactivity longer than 3 hours started the count on a new session, rather than extending the time of the current session. Use of different program components (eg, modules, tracking tool) was measured.

Clinical data were collected using brief, well-validated measures of depression (Patient Health Questionnaire [PHQ-9] [36]), anxiety (the Generalized Anxiety Disorder 7 [GAD-7] scale [37]), quality of life (European Quality of Life-5 Dimensions [EQ-5D] questionnaire [38]), and chest pain (modified Rose Angina Questionnaire [39]). The PHQ-9 [36] and the GAD-7 scale [37] are self-reported measures of severity of depression and anxiety symptoms, respectively. The PHQ-9 consists of nine items and the GAD-7 scale has seven items; for both measures, each item is scored from 0 (not at all) to 3 (nearly every day). The EQ-5D questionnaire [38] is a self-reported measure of health-related quality of life; items relating to mobility, self-care, usual activities, pain/discomfort, and mood are scored to produce a single index score ranging from 0 (worst) to 1 (best possible health). The modified Rose Angina Questionnaire [39] consists of three items designed to detect exertional chest pain which is indicative of angina; respondents report whether or not they have chest pain or discomfort "ever," "walking on the level at an ordinary pace," or "walking uphill or when hurrying." Participants were dichotomized into those who responded positively to any item and those who reported no chest pain in any circumstance. Completion rates and time taken to complete these measures were recorded in order to test their acceptability as outcome measures for a future trial.

Participants were also asked where they accessed the computer (eg, home, friend/relative’s house, public space) and whether they had any problems accessing either the computer or the intervention. Participants used Likert items and were able to enter free-text feedback concerning their satisfaction with different elements of the intervention. Within 2 weeks of them completing the trial, we contacted participants by telephone to thank them for their participation and asked if they had any further comments; if we were unable to contact them by telephone, an email was sent. Feedback collected via these methods was recorded verbatim.

#### Analysis

Descriptive statistics concerning patterns of usage, clinical measures, and their completion and responses to the satisfaction items were produced. Free-text responses (to satisfaction items and verbatim feedback data collected via telephone or email) were subject to content analysis.

## Results

### Phase 1: The Intervention

**Figure 2:**
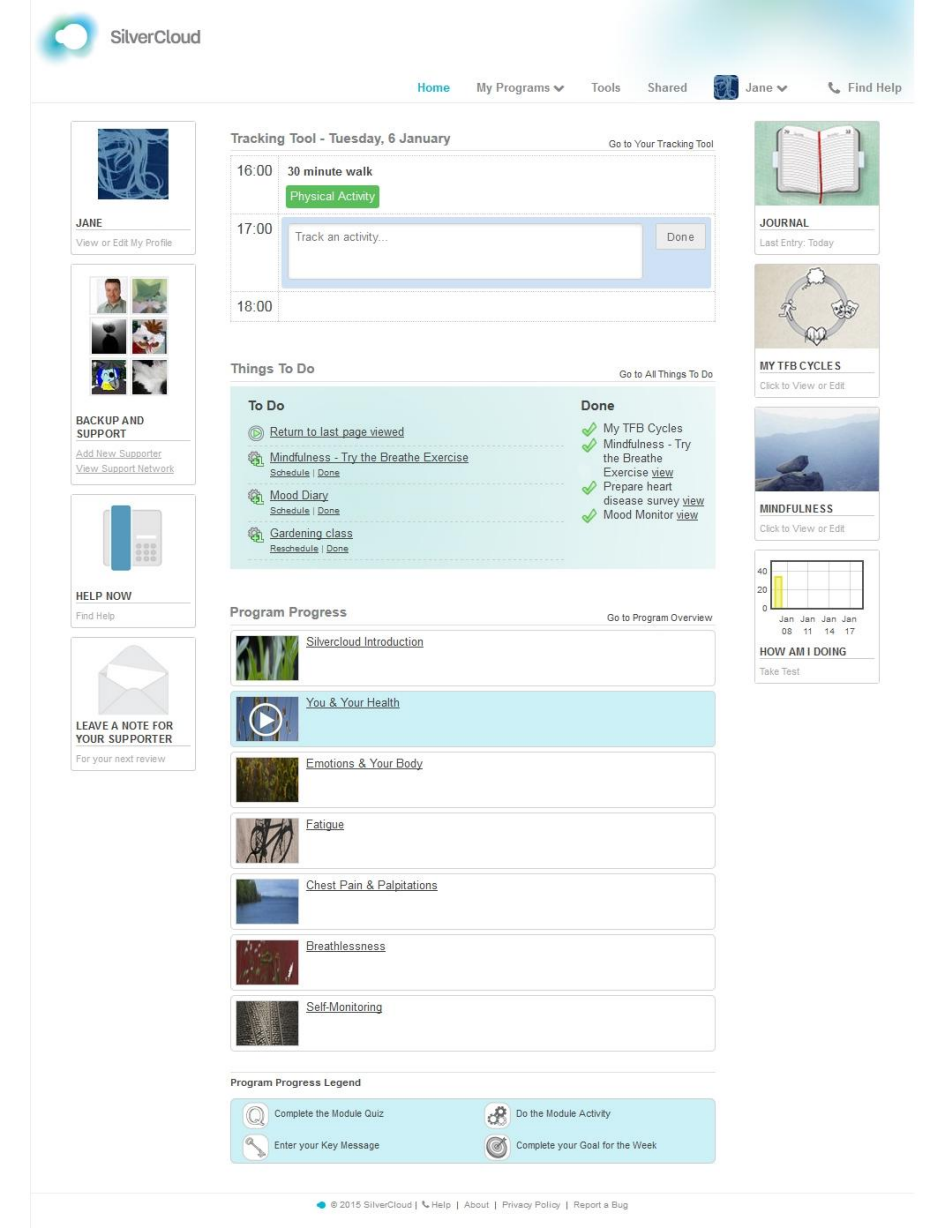
Screenshot of Space from Heart Disease home page.

Space from Heart Disease is an online psychoeducational and therapeutic intervention designed to support self-management of symptoms and to promote the well-being of people with CVD. A modular design (see Table 1) was used to increase engagement by allowing users to select content relevant to them. The existing SilverCloud intervention for distress delivers cognitive behavioral therapy; we adapted this to include CBT for coping with chest pain, fatigue, and breathlessness. Our literature review also identified specific behavior change techniques—see, for example, the Coventry, Aberdeen and London Refined (CALO-RE) taxonomy [40]—which are effective for CVD-related self-management. For instance, users are helped to apply outcome goal setting (ie, “I will drink no more than one glass of wine on week days") and action planning (ie, deciding in advance what steps to take when faced with a health-related choice, including what to do, where to do it, when to do it, and who will help) to help them to make healthy choices. A summary of module content is shown in Table 1. A tracker app allows users to record their daily activities as part of the therapy, for instance, as a homework task, and a journal function allows users to record free-text thoughts and comments. Standardized outcome measures can also be collected and results displayed graphically; quizzes are used to consolidate learning. Figure 2 shows a screenshot of the home page. Patient stories are used across the modules to provide context. The platform facilitates users to share their work, information, tasks, homework activities, etc, with a supporter, although a supporter was not provided during this development stage.

Findings from the experimental studies are reported below; how these influenced the intervention development is depicted in Figure 1.

**Table 1:** Space from Heart Disease intervention content.

Module		Module content
Self-management (You and Your Health)		Medication adherence Attending and getting the most out of appointments Promoting a healthier lifestyle: increasing exercise, healthy diet, reducing alcohol, stopping smoking, stress management
**Symptom modules**		
	1. Chest pain/palpitations	CBT^a^and behavioral strategies for coping with chest pain/palpitations
	2. Fatigue	CBT and behavioral strategies for coping with fatigue
	3. Breathlessness	CBT and behavioral strategies for coping with breathlessness
Psychological distress		Cognitive behavioral therapy for generalized distress relevant to CVD^b^
Self-monitoring (floating module linked to all others)		Guidance, support, and tools for self-monitoring

^a^Cognitive behavioral therapy (CBT).

^b^Cardiovascular disease (CVD).

### Phase 2: Focus Group Study

#### Overview

A total of 10 men were recruited from the British Heart Foundation; these represent the total number of those agreeing to participate. All participants met the inclusion criteria. No women responded to the invitation to participate in time for the discussion. Ages ranged from 53 to 85 years (mean 70 years, SD 11), all participants were Caucasian, 5 of the 10 (50%) were in current employment, and 5 (50%) were retired. Out of the 10 participants, 1 (10%) left school at 16, 4 (40%) had further education qualifications, 3 (30%) had undergraduate degrees, and 2 (20%) had postgraduate degrees. Participants reported one or more of the following: coronary stent inserted (4/10, 40%), coronary artery bypass surgery (4/10, 40%), comorbid type 2 diabetes (2/10, 20%), and chronic kidney disease (1/10, 10%). The discussion lasted 104 minutes.

Four key themes were identified from the discussion: information needs, physical health concerns, mental health impacts, and self-management. Themes are described with supporting quotations identified by participant number (eg, Participant #1 to Participant #10). All participants contributed to all themes and there appeared to be strong consensus within the group.

#### Theme: Information Needs

Participants wanted information to be presented clearly and to be jargon free. Some participants wanted a lot of information, whereas others preferred to have key facts:

Some people are really interested in the full story and others like me would be interested in broad guidance

Participant #3

The importance of treating users as individuals was noted:

The way you interpret it and manage your life varies absolutely as an individual.

Participant #8

It needs to be that I can choose I’m not interested in that bit, I’m not interested in that bit, that bit’s really relevant to me, and build my own package up from what’s available in there.

Participant #4

They also wanted to be confident that the information provided was reliable and represented the best advice. There was consensus that there is so much, often conflicting, information available that it can be hard to know what to read:

If I type how to avoid type 2 diabetes into Google I will get probably some millions of pieces of advice.

Participant #8

You get conflicting advice from people...everyone reckons they’re right don’t they, so how do I know you’re going to be right?

Participant #3

There was consensus that no existing resource met all their information needs:

The difficulty is you do the expert course which just looks at your diabetes and you do the post-operative cardiac care which just looks at your cardiac care and there’s nobody ever puts the two together.

Participant #6

There was also consensus that ongoing support is required:

At the end of that eight weeks [of cardiac rehabilitation classes] I was suddenly told, sorry, finished now, off you go and it was like having the umbilical cord cut, like what’s going to happen now?

Participant #9

The data from this theme led us to include "expand and collapse" options within the intervention so that users can select the amount of detail they wish to read. These findings supported the modular design of the intervention whereby users can choose which aspects of the intervention to access according to their needs. We also made the intervention as comprehensive as possible and provided links to reputable websites.

#### Theme: Physical Health Concerns

On the whole, the participants, who were all active members of a BHF support group, did not report being troubled by physical symptoms:

We’ve learnt to manage it.

Participant #5

They were very concerned, however, about the possibility of having a (another) heart attack. They were aware of each other’s experiences and knew that each was different:

The confusion is that different individuals feel completely different symptoms...X had terrible pain, I had no pain whatsoever and suddenly I collapsed.

Participant #2

Even those members who had had more than one cardiac event agreed that no two events felt the same. Participants agreed that they would like information to help them know when to seek help:

I’m a little bit confused about people talking about heart attack and angina...when you’re getting the pain, which is which?

Participant #1

I’d like to see something...which would tell me, is this normal or not, or should I worry?

Participant #6

Following identification of this theme, we added advice for participants about when to seek help.

#### Theme: Mental Health Impacts

Participants discussed the impact of CVD on mental health:

What I found with people who have heart problems and who have operations or whatever, they have to live with their mortality on a day to day basis.

Participant #5

...having been fit and thinking I was going to live forever basically, that I then was diagnosed with obviously heart disease then I’m now being told I’m borderline diabetic sort of thing which is another one I do not want to hear. And I don’t think the medical profession really appreciate, well we’ll tell him and we’ll give him all this information and he can go away and he’ll be all happy to sort it out. Well I’m not happy really, I didn’t want to be diagnosed.

Participant #3

Though not currently depressed, several participants reported past low mood associated with cardiac events:

I did suffer depression because I couldn’t get my (driving) license back so I wasn’t working for a year and a half.

Participant #4, taxi driver

For about two weeks during my recovery program whenever I spoke to anybody about my experiences...I just couldn’t stop crying. And this lasted for about two weeks. I didn’t want to, I didn’t feel like I wanted to cry, tears just came.

Participant #9

It took six years for me to go through the post op depression.

Participant #5

However, other participants appeared not to want to dwell on this topic and moved the discussion on. It is unclear what help people had received for their low mood; that some reported long durations suggests that effective help had not been available. We have ensured that help for low mood and anxiety is woven throughout the modules.

#### Theme: Self-Management

These participants agreed with the importance of self-management and that a heart attack could trigger positive lifestyle changes:

...when you walk out of the hospital and you’ve done the rehab, well the word I use is you’re surviving, you’re no longer a patient, you’re responsible for your own health.

Participant #8

...the best thing that can happen to you is to have a heart attack because it makes you think about your health.

Participant #1

As well as belonging to a support group, which they found helpful, some participants used devices to help them to live more healthily:

I mean X’s got Fitbit and I’ve got the same sort of thing and you get an email from them once a week and you can open it if you like and if you open it, it tells you how many steps you’ve done in the last week, how many times you’ve been upstairs, it’s just a reminder, it’s not particularly intrusive but it actually gives you the feeling that you are in a certain sense monitoring your own health.

Participant #8

Participants liked the idea of an intervention which could prompt them to make healthy choices and monitor their progress:

If there was a program which helped me monitor the sort of things that I ought not to do just to stay off, you know, I’ve been warned about it and I think that I’m okay, but just to be reminded etc and to track my own progress which means I might set some simple objective for myself.

Participant #8

However, there was consensus that they did not want to spend too much time using an intervention or on self-management activities:

I don’t want to spend my retirement filling in things and trying to work out whether I’m all right or not.

Participant #3

...the thing is to lighten the load, that’s why I say if you have a to-do list have a to-don’t list because the fact is we’re not going to do all the things on the to-do list or stick to all the resolutions.

Participant #8

The You and Your Health module provides tools for making healthy choices. The intervention is flexible to allow individuals to spend as much or as little time as they choose using it.

### Phase 3: Think-Aloud Usability Testing

One male (aged 60 years) and one female (aged 50 years) each spent 60 minutes using the intervention. Participants were Caucasian, one left school at 16, the other had an undergraduate degree, and both reported having CHD. Overall, the two participants liked the intervention, they continued to use it until the session ended, and were able to navigate without difficulty. User comments and researcher observations are summarized in terms of strengths and weaknesses in relation to "readability," "information," and "navigation," and are shown in Tables 2 and 3, respectively. The intervention was adapted accordingly (see Figure 1). Minor bugs were also noted and fixed.

**Table 2:** Usability strengths identified in think-aloud testing.

Intervention characteristic	Usability strengths	
	Participant 1	Participant 2
Readability	N/A^a^	Easy to understand
Information	Relevant	Relevant
Navigation	Found commenting easy Used "like" buttons a lot Easy to move between modules Used the side menu and the next/previous buttons without difficulty Easy to select relevant information to read	Arrows could clearly be clicked Used side menu and next/previous buttons without difficulty Opened expandable subtopics easily True/False buttons easy to use
Bugs	N/A	N/A

^a^Not applicable (N/A). Participant did not comment on this aspect of the intervention.

**Table 3:** Usability problems identified in think-aloud testing.

Intervention characteristic	Usability problems	
	Participant 1	Participant 2
Readability	Font too small in some places	Font too small in some places Links and icons should be bigger Too much text in some sections
Information	Would like more links (eg, to charities, support services) More content could be given in some places, using a "tiered approach"	Would like more links (eg, to charities, psychological services) Add dietary information for diabetic patients and vegetarians Provide more information on medications
Navigation	No problems	"Dots" were confusing (didn’t realize they could be clicked) Tried to click on some icons, they look too much like buttons In self-monitoring module, didn’t realize the subtopics were support content
Bugs	Difficult to remove comments "Liking" twice (because of liking something very much) made it "un-like"	N/A^a^

^a^Not applicable (N/A). Participant did not comment on this aspect of the intervention.

### Phase 4: Cross-Sectional Study

#### Overview

A total of 10 participants (2 female, 20%) were recruited; 6 (60%) had participated in the focus group, 1 (10%) had participated in both the focus group and the think-aloud test, 1 (female, 10%) had participated in the think-aloud test, and 2 (20%) (1 female) were recruited via snowballing. Participants were aged from 65 to 85 years (mean 70 years, SD 9). All participants were Caucasian; out of 10, 4 (40%) had left school at age 16, 3 (30%) had an undergraduate degree, and 3 (30%) had a postgraduate degree. Out of 10, 5 (50%) participants were retired, 3 (30%) were working full time, and 2 (20%) were working part time. Self-reported confidence in using the Internet was as follows: out of 10, 2 (20%) participants were "very confident," 3 (30%) were "confident," 2 (20%) were "mildly confident," and 3 (30%) reported "average" confidence. Out of 10, 7 (70%) participants reported having had at least one cardiac event. None reported current depression or anxiety, but in the past, 4 out of 10 (40%) participants had received psychotherapy and/or medication for these conditions.

#### Usage

All participants reported using their home computers with no access difficulties with either the computer or the intervention. Over the 2-week testing period, there was considerable variation in the number of occasions and amount of time spent using the intervention. The average number of sessions per user was 2.4 (SD 2.2, range 1-8) and the mean time per session was 23 minutes (SD 15, range 5-65). All users also had one or more log-ins of less than 5 minutes (range 1-8). The total time spent using the intervention per participant ranged from 8 to 197 minutes (mean 57, SD 59). All aspects of the intervention were accessed: 43% of time was spent using the content, 26% of time was spent using apps on the platform, 16% of time was spent browsing the home screen, 3% of time was spent using the journal, and 12% of time was spent completing the clinical outcome measures.

#### Clinical Outcomes

At baseline, all 10 (100%) participants completed all four outcome measures—PHQ-9, GAD-7 scale, EQ-5D questionnaire, and the modified Rose Angina Questionnaire—and at the end of 2 weeks, only 5 (50%) participants returned data for these measures. Measures took a maximum of 1 minute and 9 seconds to complete. There were no missing answers. At baseline, 4 out of the 10 (40%) participants reported current chest pain (modified Rose Angina Questionnaire [39]) and at 2 weeks, 2 out of 5 (40%) participants who responded reported chest pain. Questionnaire scores indicated that the participants were not depressed or anxious and had a good quality of life (see Table 4).

**Table 4:** Participant ratings of depression, anxiety, and quality of life from the cross-sectional study (n=10).

Clinical outcome	Median (IQR^a^)		Range	
	Baseline	2 weeks	Baseline	2 weeks
Depression (PHQ-9^b^)	2 (3)	0 (2)	0-8	0-6
Anxiety (GAD-7^c^)	2 (3)	0 (0)	0-7	0-5
Quality of life (EQ-5D^d^)	0.95 (0.5)^e^	0.90 (0.4)^e^	0.50-1	0.50-1

^a^Interquartile range (IQR).

^b^Patient Health Questionnaire (PHQ-9). Scoring range for the PHQ-9 was 0-27 (depression scores: 0-4 minimal, 5-9 mild, 10-14 moderate, 15-19 moderately severe, 20-27 severe).

^c^Generalized Anxiety Disorder 7 (GAD-7). Scoring range for the GAD-7 scale was 0-21 (anxiety scores: >5 mild, >10 moderate, >15 severe).

^d^European Quality of Life-5 Dimensions (EQ-5D). Scoring range for the EQ-5D questionnaire was 0-1 (quality-of-life scores: 0 worst, 1 best possible health).

^e^For this measure, there were 5 participants.

#### Satisfaction

Out of 10 participants, 8 (80%) responded to the questions concerning satisfaction with the intervention. Overall, 7 out of 8 (88%) reported being "satisfied" with the intervention and 1 out of 8 (13%) reported being "very satisfied"; 7 out of 8 (88%) would recommend the intervention to someone else. Participant responses to the intervention use and satisfaction items are shown in Tables 5 and 6, respectively.

**Table 5:** Participant responses to intervention use items (n=8).

Intervention use items	Yes, n (%)	No, n (%)	Not sure, n (%)
Easy to use	8 (100)	0 (0)	0 (0)
Informative	8 (100)	0 (0)	0 (0)
Helpful with any difficulties you are having	5 (63)	2 (25)	1 (13)
Any changes in any area of your life since using intervention^a^	1 (14)	6 (86)	0 (0)
Would you recommend intervention?	7 (88)	1 (13)	0 (0)

^a^This item had one missing value (n=7).

**Table 6:** Participant responses to satisfaction items (n=8).

Satisfaction with intervention	Very satisfied, n (%)	Satisfied, n (%)	Somewhat satisfied, n (%)	Somewhat dissatisfied, n (%)	Dissatisfied, n (%)
Overall	1 (13)	7 (88)	0 (0)	0 (0)	0 (0)
You and Your Health (self-management module)	0 (0)	7 (88)	1 (13)	0 (0)	0 (0)
Emotions and your body module	0 (0)	8 (100)	0 (0)	0 (0)	0 (0)
Fatigue module	0 (0)	5 (63)	2 (25)	1 (13)	0 (0)
Chest pain module^a^	0 (0)	5 (71)	2 (29)	0 (0)	0 (0)
Breathlessness module	0 (0)	5 (63)	2 (25)	1 (13)	0 (0)
Tracker app (self-monitoring)	0 (0)	6 (75)	1 (13)	1 (13)	0 (0)

^a^This item had one missing value (n=7).

#### Content Analysis of Free-Text Feedback

Participants were asked to "use 3 words to describe the intervention" (quotes in this section are representative examples). Out of 10, 5 (50%) people’s responses suggested they found the intervention easy to use (eg, "easy," "piece of cake," and "straight forward"), 3 (30%) others focused on the usefulness of the intervention (eg, "helpful, useful, informative"), and 1 (10%) person each described it as "reassuring," and "focused or clear." The only negative words were from 1 (10%) person, who described the intervention as "static, impersonal," but also "good quality."

A final person (1/10, 10%) said "not necessarily relevant." This person also sent a long email explaining that they had had their heart attack many years ago and felt that their condition was under control; they did not enjoy filling in the depression and anxiety measures and felt that the intervention was too focused on low mood for someone like himself.

The aspects of the intervention which were most liked included the presentation (“The screens are nicely laid out.” [Participant #1]), clarity of information (“What was being asked was clear and the information was clear.” [Participant #3]), ease of use (“easy to use” [Participant #5]), and the ability to choose relevant aspects of the intervention (“Self-selection helpful as I really only have problems with fatigue and how to deal with it.” [Participant #9]).

Aspects least liked included diagrams being “too busy” [Participant #2], “emphasis on depression” [Participant #3], and “uninspiring case studies—however, I can see how they might help others” [Participant #7]. One would have liked “more in-depth information” [Participant #9] and one “didn’t dislike anything” [Participant #10].

Out of 10 people, 4 (40%) said that an online supporter (as planned) would be useful.

Only 1 (10%) participant had used an online heart-related website before. In comparison to that (unknown) website, they reported that Space from Heart Disease was “Much better in terms of content, use and appearance. Not as good in terms of interactivity or helping a person achieve changes in life-style.” [Participant #1].

Some participants did not think the intervention was useful to them personally as they were not experiencing the mood or symptom problems that it addressed.

## Discussion

### Principal Findings

This paper describes the development and preliminary acceptability and feasibility of a new CVD-specific online intervention to promote self-management and well-being: Space from Heart Disease. The development process was informed by MRC guidelines for the development of complex interventions [29]. This enabled us to produce an intervention which builds on existing evidence, is theory based (ie, CBT and behavior change theory), and is flexible, personalized, and service-user informed.

For instance, the focus group participants varied in their information needs and said they wanted content to be delivered flexibly in order to increase their control in using the intervention. This feedback led to the use of "expand and collapse" options where users can "click" to see information which is hidden from those who find too much text off-putting. The use of modules allows people to skip sections not relevant to them, for instance, only people experiencing chest pain would use the chest pain module. In our cross-sectional study, only 4 people reported chest pain at baseline; future research will recruit people with CVD symptoms in order to test the efficacy of Space from Heart Disease. The symptoms currently covered by Space from Heart Disease are those which have been found in research to cause impaired quality of life and increased unnecessary health care usage [41,42]; if further problematic symptoms are identified, modules to address them could be developed. Specific information that was added following feedback included how to recognize a heart attack, when to seek medical help, and information concerning diabetes, which is common in people with other CVD-related conditions [43].

Following the focus group, adaptations were made to the intervention and we conducted think-aloud usability testing. This was a useful stage in the development process as it highlighted potential improvements relating to readability (eg, font size too small) and navigation (eg, confusion around which features were "clickable") that could not have been tested in the focus group. A difficulty in removing comments should the participant change their mind was also highlighted. Using several evaluation methods, therefore, helped us to identify a wider range of potential problems; the benefits of this approach have been noted previously [44].

The Space from Heart Disease intervention builds on a generic online intervention for depression and anxiety that is currently used within the NHS. Space from Heart Disease includes a module on distress, which patients may or may not select, which highlights CVD-related dysfunctional thinking (ie, "catastrophizing"—assuming that chest pain is always serious, leading to panic and inappropriate responses such as avoidance of activity, which can lead to worse health and unnecessary health service use) [45]. However, the impact of low mood and anxiety is stressed throughout the intervention; feedback from the cross-sectional study of participants suggests that people who are not currently experiencing distress may not consider the intervention relevant to them. A cohort study of 548 people with CHD [46] found that 22% of participants reported that life was better since their diagnosis supports this. Those participants reported that diagnosis of CHD had led to healthy lifestyle changes, reduction of stress, and recognition of their mortality; such people would be unlikely to benefit from Space from Heart Disease. This suggests that future research to test the effectiveness of Space from Heart Disease should target only CVD patients who report comorbid distress; studies such as one that tested a telephone intervention for primary care patients with CHD and distress [47] indicate that this is feasible.

It is sometimes assumed that older or less-educated people will not be able to access or use technology. In this work we recruited people with a range of educational backgrounds and self-reported levels of confidence using a computer; an important finding was that no participant reported problems either using or accessing the intervention. This is a preliminary indication of the feasibility of the intervention in this population.

Finally, the focus group data indicated that users would appreciate a tailored approach. In future, we plan to develop a supporter role to facilitate the intervention. The original SilverCloud intervention employs such a supporter who is able to provide tailored encouragement and feedback, for instance making suggestions regarding which elements of the intervention the user may find helpful. Future work will determine what skills are needed for a supporter of a CVD-specific intervention—that is, whether medical or psychological skills are most needed. Candidate professionals to fulfil this role may be nurses or psychological well-being practitioners as employed within the UK Improving Access to Psychological Therapies service.

### Strengths and Limitations

Informed by the MRC guidelines for the development of complex interventions [29], we used multiple research designs to develop and make systematic, service-user informed improvements to a new CVD-specific intervention for the promotion of self-management and well-being. To our knowledge, Space from Heart Disease is the first online intervention in CVD which addresses self-management, symptom management, and psychological distress. Use of the MRC guidelines [29] enabled us to develop content which builds on existing research and uses evidence-based CBT and behavior change techniques. We used the existing SilverCloud online platform for delivering CBT as the basis of our CVD-specific intervention. The SilverCloud intervention was developed with extensive user input and is known to be effective for improving psychological distress [30-32]. Our approach, therefore, differs from that of others who have used focus group data as the basis of their intervention. For instance, Antypas and Wangberg [48] conducted a focus group to determine user needs and combined findings with a theoretical review of health behavior models to develop a novel online intervention for physical activity in cardiac rehabilitation.

Our sample sizes were small, so the participants may not be representative of the larger population of people with CVD and, in particular, the views of women are lacking since only two were recruited. Our use of the large body of existing literature on distress and symptoms in CVD was designed to ensure that Space from Heart Disease meets a range of needs, however, the needs of women are often neglected in CVD research [49] and in future, researchers should make additional efforts to address this. Future trials of Space from Heart Disease will determine whether there was sufficient input from the range of people with CVD to produce an effective intervention. Nevertheless, the level of patient and public involvement in the development of the intervention is high as adaptations to the intervention were made as a direct result of in-depth participant feedback. Our use of a series of studies using different designs ensured that all aspects of the intervention were tested.

Our cross-sectional study provides preliminary support for the acceptability and feasibility of Space from Heart Disease in people with CVD—patients did not report problems using the intervention and feedback indicated that the content was felt to be relevant. However, none of our participants was currently experiencing depression or anxiety, so further work is needed to test the acceptability of the intervention in people with CVD and comorbid psychological distress using a much larger sample. Space from Heart Disease builds on an existing, effective, generic intervention for depression and anxiety which is designed to be facilitated by a professional "supporter" who provides timely and personalized feedback; future research will test the effectiveness of Space from Heart Disease delivered with online support.

### Conclusions

Health information technology can improve health care quality and safety [50,51]. Online-delivered therapy is effective for psychological distress [19]. However, generic computerized CBT packages may not be acceptable for people with comorbid physical health problems [23]. We have developed an evidence-based, theory-informed, user-led online intervention for improving self-management and well-being in CVD. The use of multiple evaluation tests has informed improvements to its content and usability, and preliminary acceptability and feasibility have been demonstrated. This is important since the effectiveness of online interventions may be hampered by design and usability problems [44]. This development work also identified the most appropriate sample (ie, people with symptomatic CVD and comorbid distress) for a future RCT of the effectiveness of Space from Heart Disease.

## Appendix

Multimedia Appendix 1 Topic guide for focus group.

## Abbreviations

BHF: British Heart Foundation
CALO-RE: Coventry, Aberdeen and London Refined
CBT: cognitive behavioral therapy
CCTV: closed-circuit television
CHD: coronary heart disease
CVD: cardiovascular disease
EQ-5D: European Quality of Life-5 Dimensions
GAD-7: Generalized Anxiety Disorder 7
IAPT: Improving Access to Psychological Therapy
IP: intellectual property
IQR: interquartile range
KCL: King’s College London
LTC: long-term condition
LTC/MUS: long-term conditions/medically unexplained symptoms
MRC: Medical Research Council
MUS: medically unexplained symptom
N/A: Not applicable
NHS: National Health Service
NICE: National Institute for Health and Care Excellence
NIHR: National Institute for Health Research
PHQ-9: Patient Health Questionnaire
RCT: randomized controlled trial
