# Supplementary material for: The Space From Heart Disease Intervention for People With Cardiovascular Disease and Distress: A Mixed-Methods Study
Source: JMIR Res Protoc. 2015 Jul 1;4(3):e81. doi: 10.2196/resprot.4280 (PMC4526970; doi:10.2196/resprot.4280)
Supplement: Multimedia Appendix 1 [file resprot_v4i3e81_app1.pdf]

Appendix A: Topic Guide

Development, Feasibility and Acceptability of the 'Space from Heart Disease' Intervention for people Cardiovascular Disease and Distress: a mixed methods study.

Welcome & Introductions:

- The reason we are here is to discuss problems with having a heart-related disease and what would help you to manage better.
- We are developing an online resource to help people with heart-related diseases, we will show you an example online resource and will ask your opinions on it.
- You were selected because you have told us that you have a heart-related disease.

Guidelines:

- No right or wrong answers, only differing points of view
- We're tape recording, one person speaking at a time
- We're on a first name basis here but when we write up the report we will ensure that your identities remain confidential
- You don't need to agree with others, but you must listen respectfully as others share their views
- We ask that you turn off your phones for the duration of the session. If you cannot and if you must respond to a call please do so as quietly as possible and rejoin us as quickly as you can.
- My role as moderator will be to guide the discussion, please talk to each other

Topics

Some people with heart-related diseases say they have chest pain or palpitations, fatigue and or breathlessness and that their condition makes them feel stressed.

- Do you, or anyone you know, have experience of any of these?
- How do these make you feel?
- How do you or anyone you know, cope with these things?

You may be aware that being overweight, drinking too much and not exercising can be bad for heart disease.

- What helps or hinders you from having a healthy lifestyle?

Many people with heart-related diseases have to take a lot of medications

- Do you, or anyone you know, have problems with taking medications?

Demonstration of SilverCloud example intervention

We would now like to show you an example of the kind of online resource we would like to develop. This one is about depression; the one we develop will be specific to heart-related diseases.

Run through application and ask for comments on each element: (prompts – more/less required; language ok, any changes?)

Video

Quiz

Information pages etc

Do you think a similar resource more focused on heart-related diseases would be useful to you?

Demonstration of Managing CVD and CVD distress modules (printed copies)

These are examples of the kinds of heart related material which will be online – please read, any comments?

Is there anything you can think of which we should include in the resource that we haven't already covered?

Personal stories

Finally, we will be illustrating the programme using examples from these people's personal stories. Please let us know what you think of each one.

Thank you very much for your thoughts, ideas and reflections
